# Characterization of the complete chloroplast genome of *Salix gordejevii* (Salicaceae)

**DOI:** 10.1080/23802359.2021.1959438

**Published:** 2021-07-27

**Authors:** Yinghao Wei, Xiaoping Li

**Affiliations:** a Key Laboratory of Forest Tree Genetics and Breeding and High-Efficiency Cultivating in Jiangsu Province, Nanjing, China; b College of Forestry of Nanjing Forestry University, Nanjing, China; c Poplar Germplasm Resource Nursery in Jiangsu Province, Nanjing, China

**Keywords:** Chloroplast genome, *Salix gordejevii*, Salicaceae, Phylogenetic tree

## Abstract

*Salix gordejevii* is an endemic species in northern China. The analysis of the complete chloroplast genome of *S. gordejevii* can offer a referential basis for identifying and utilizing *Salix* germplasm resources. In this study, we obtained chloroplast DNA from *S. gordejevii* and characterized it. The complete chloroplast genome of *S. gordejevii* is 155,491 bp in length, comprising a pair of inverted repeats (IR, 27,408 bp), a large single-copy region (LSC, 84,367 bp), and a small single-copy region (SSC, 16,309 bp). We annotated 117 genes in total, including 80 protein-coding genes, 32 tRNA genes, four rRNA genes, and one pseudogene (ycf1). A maximum-likelihood phylogenetic tree was built with MEGA-X and showed that the chloroplast of *S. gordejevii* has the closest relationship with that of *S. magnifica* compared to the other reported *Salix* genomes.

The chloroplast is the core organelle in photosynthesis. Due to its high conservation, the chloroplast genome is widely utilized in species identification and phylogenetic analyses (Szymon et al. 2016). The sequencing and analysis of the chloroplast genome are also of great significance in improving plant traits and photosynthetic efficiency (Chen and Liu 2008). *Salix gordejevii,* which belongs to the genus *Salix* in the family Salicaceae, is widespread in eastern Inner Mongolia, western Liaoning, Gansu, and the Ningxia Hui Autonomous Region, China (Liu 1984). It is an important tree species for afforestation and desertification control in the northern region of China due to its strong drought resistance (Cao et al. 2018). Additionally, *S. gordejevii* has become the preferred biological sand barrier for sandstorm source control projects in the dune areas of Beijing-Tianjin (Ma et al. 2019). Currently, the complete chloroplast genome sequence of *S. gordejevii* remains to be sequenced and analyzed, and it can serve as a referential basis to assess the phylogenetic relationships among 24 previously reported *Salix* species and reconstruct reticulate evolution. In this study, we report the chloroplast genome of *S. gordejevii* and reconstruct a phylogenetic tree for further analysis.

Fresh leaves of *S. gordejevii* were collected from the Ningxia Hui Autonomous Region (37.736692°N, 107.348077°E). The voucher specimen (voucher number: NXHL2017002) was deposited at the herbarium of Nanjing Forestry University (https://linxue.njfu.edu.cn/, Xiaoping Li, xpli@njfu.edu.cn). A modified CTAB method was adapted for the extraction of genomic DNA (Doyle and Doyle 1987). DNA fragment libraries were constructed with an average insert size of 300 bp and then sequenced using the Illumina NovaSeq 6000 platform (Illumina, San Diego, CA). After removing flanking sequences and low-quality sequences with Fastp (Chen et al. 2018), 5.1 GB of high-quality clean reads were assembled with NOVOPlasty (Dierckxsens et al. 2017). The assembled chloroplast genome was annotated using GeSeq (Tillich et al. 2017) (https://chlorobox.mpimp-golm.mpg.de/geseq.html) with the chloroplast genome of *S. dasyclados* as a reference (GenBank accession number: MT551160), and the annotated genome was corrected by manual curation. The annotated cpDNA sequence of *S. gordejevii* was uploaded to GenBank (GenBank accession number: MW562004).

The annotation results indicate that the chloroplast genome of *S. gordejevii* is 155,491 bp and has a typical quadripartite circular structure. Similar to the chloroplast genome of most higher plants, there are two inverted repeat regions (IRB and IRA, 27,408 bp), a large single-copy region (LSC, 84,367 bp), and a small single-copy region (SSC, 16,309 bp). The CG content of the chloroplast genome is 36.7%. In total, 117 genes were annotated, including 80 protein-coding genes, 32 tRNA genes, four rRNA genes, and one pseudogene.

For the phylogenetic analysis of *S. gordejevii* and other *Salix* species, a total of 25 chloroplast genomes of Salicaceae were downloaded from NCBI. MAFFT 7 (https://mafft.cbrc.jp/alignment/server/) (Katoh et al. 2002) was used to align the sequences. A maximum-likelihood phylogenetic tree (with 1000 bootstrap replicates) was built with a general time reversible (GTR) nucleotide substitution model using the MEGA-X program (Tamura et al. 2013), in which *Populus alba* served as the outgroup. The bootstrap support value (%) is shown next to the nodes. As shown in the phylogenetic tree (Figure 1), *S. gordejevii* was most closely related to *S. magnifica*. The newly annotated plastome of *S. gordejevii* will be helpful for the phylogenetic identification and utilization of *Salix* germplasm resources. 

**Figure 1.**
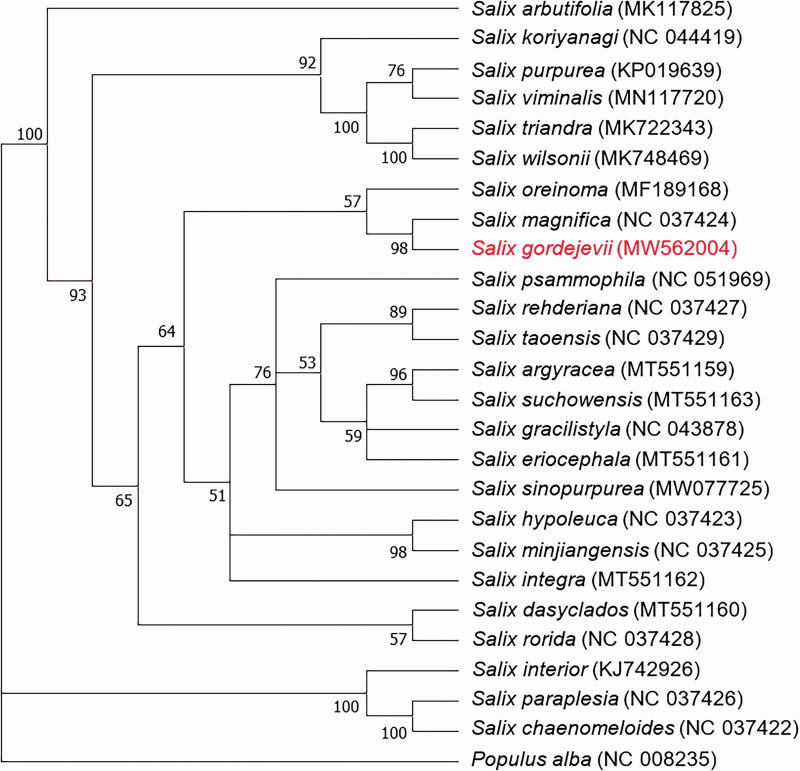
Phylogenetic tree based on 26 chloroplast genome sequences with 1000 bootstrap replicates. The bootstrap support value (%) is shown next to the nodes.

## Data Availability

The genome sequence data that support the findings of this study are available in GenBank of NCBI (https://www.ncbi.nlm.nih.gov/) under the accession number MW562004. The associated BioProject, SRA, and Bio-Sample numbers are PRJNA734857, SRR14718608, and SAMN19547117 respectively.
